# Randomized, Double-Blind, Placebo-Controlled Study to Assess the Efficacy of Nonopioid Analgesics on Pain following Arthroscopic Knee Surgery

**DOI:** 10.1155/2012/305821

**Published:** 2012-11-05

**Authors:** Susanne Abdulla, Regina Eckhardt, Ute Netter, Walied Abdulla

**Affiliations:** ^1^Department of Anesthesiology and Intensive Care Medicine, Klinikum Bernburg, Martin Luther-University Halle-Wittenberg, Kustrenaer Straße 98, 06406 Bernburg, Germany; ^2^Department of Neurology, Otto-von-Guericke University, Leipziger Straße 44, 39120 Magdeburg, Germany; ^3^Department of Neurology, Medizinische Hochschule Hannover, Carl-Neuberg-Straße 1, 30625 Hannover, Germany; ^4^Department of Anesthesiology, Johannes Gutenberg University Mainz, 55131 Mainz, Germany

## Abstract

*Purpose*. In a randomized, double-blind trial, the efficacy of nonopioid analgesics on postoperative piritramide consumption was compared for pain relief during the first 24 h in patients recovering from arthroscopic knee surgery. *Methods*. 120 patients were treated with normal saline and/or one of the nonopioid analgesics (parecoxib, metamizole, paracetamol) in addition to piritramide using the PCA pump. Beginning in the postanesthesia care unit (PACU), patients were asked to quantify their pain experience at rest while piritramide consumption was recorded. *Results*. Piritramide consumption upon arrival in the PACU was high in all groups. However, cumulative consumption in the parecoxib group was significantly lower than that in the placebo group at 6 and 12 h after surgery. At discharge from the PACU, VAS scores dropped in all groups and were significantly lower in the parecoxib group. In the PACU, satisfaction of the patients was moderate and improved with time after surgery. *Conclusions*. There was statistically significant opioid-saving effect by administering parecoxib with better VAS scores and satisfaction level compared to placebo. The high pain score in the PACU in all groups immediately after recovering from remifentanil-based anesthesia would be prevented if local anesthetics were administered intra-articularly as part of a multimodal analgesic approach.

## 1. Introduction

Arthroscopic knee surgery belongs to those procedures with the highest incidence of moderate to severe pain for the first 24 hours postoperatively [1]. A variety of analgesic drugs and techniques with controversial results have been used to relieve pain after arthroscopic knee surgery [2]. Recently, it was advised to use systemic analgesics instead of intra-articular bupivacaine or ropivacaine for pain relief after knee arthroscopy [3]. The multimodal analgesic technique with a combination of opioids using a PCA pump and non-opioid-analgesics with differing mechanisms of action is still a very popular approach to prevent pain after surgery, and the opioid-sparing effect may lead to reduced nausea, vomiting, urinary retention, respiratory depression, and sedation [4–6].

Although the majority of PCA studies was conducted with morphine [7–12], only few studies have evaluated the consumption of piritramide after different surgical procedures [13–16]. Piritramide has been used in parts of Europe and South America as the analgesic opioid of choice for the management of postoperative pain for more than 40 years [13]. Its relative analgesic potency compared with morphine is approximately 0.7, and its duration of action lasting for 4 to 6 hours is relatively long because it is distributed extensively and eliminated slowly [17]. With piritramide, hemodynamic changes are not expected to occur; however, differences in the incidence of vomiting during PCA therapy between the two mu-receptor agonists piritramide and morphine were not found [18, 19].

The selected non-opioid analgesics including parecoxib, metamizole (dipyrone), and paracetamol are routinely used for the IV treatment of postoperative pain in Germany. Both parecoxib and metamizole are considered nonsteroidal anti-inflammatory drugs (NSAIDs), albeit one is a selective COX-2 inhibitor while the other is not selective. Parecoxib is the only parenterally administered selective COX-2 inhibitor which has the most supportive data for its beneficial effects as a part of multimodal analgesia and offers benefits with regard to its adverse effect profile [20, 21]. Metamizole is still widespread used in Europe and South America. In addition to its analgesic properties, it has antispasmodic and antipyretic effects, particularly in patients with visceral pain. In other countries it is banned because of an association with life-threatening blood agranulocytosis although the strength of the association has been a matter of much debate [22–27]. However, in the discussion of metamizole-induced agranulocytosis, the overall risk of NSAIDs with regard to their potentially life-threatening adverse effects should be considered in comparison with other nonopioid analgesics which are not devoid of serious side effects [28]. Nevertheless, because of the risk of agranulocytosis after metamizole (for long-term use), patients should be monitored for blood dyscrasias and, extremely rarely, broad-spectrum antibiotics with hematopoietic growth factors be administered if agranulocytosis occurs [15]. Paracetamol, on the other hand, is as para-aminophenol in a different chemical class and not considered anti-inflammatory. It can now also be administered intravenously and has thus gained renewed interest in this setting due to its minimal adverse effects.

Since there is obviously no randomized study that compares IV administered parecoxib versus metamizole (dipyrone) or paracetamol (acetaminophen) on piritramide consumption in the early postoperative period of arthroscopic knee surgery, the aim was therefore to perform a prospective, randomized, double-blind, placebo-controlled study in patients undergoing arthroscopic knee surgery. The primary objective was to compare postoperative piritramide consumption alone or in combination with parecoxib, metamizole, or propacetamol (paracetamol) for providing pain relief in adult patients recovering from arthroscopic knee surgery during the first 24 hours. Secondary outcomes included pain intensity and patient satisfaction.

## 2. Patients and Methods

The investigative protocol was approved by the institutional review board at our teaching hospital on December 9, 2003, and all patients provided written informed consent before enrolment. The study began in March 2004 and ended in August 2007. Inclusion criteria were the following: patients in the age between 18 and 75 years and ASA physical status I–III who underwent specific arthroscopic knee surgery under tourniquet. Patients undergoing ambulatory knee surgery as well as patients with a history of significant cardiac, pulmonary, hepatic, or renal disease, morbid obesity, chronic pain and drug or alcohol abuse, and contraindications or previous adverse reaction to any of the drugs used in the study were excluded. Also not included were patients unable to cooperate.

**Figure 1:**
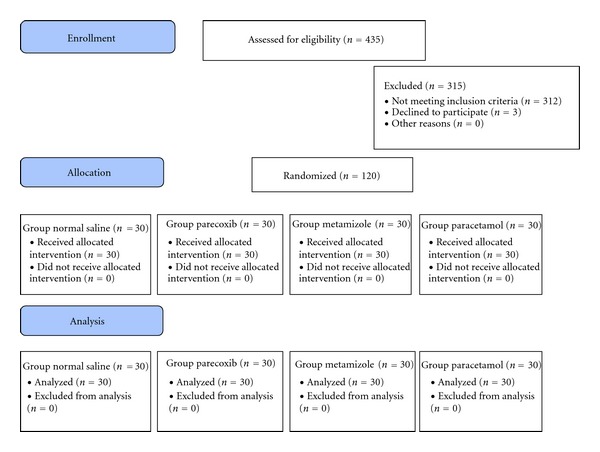
The CONSORT flow diagram of the study design for arthroscopic knee surgery study.

Patients meeting the inclusion criteria and scheduled for arthroscopic knee surgery under general anesthesia were pre-admitted the night prior to surgery, and the use of PCA for postoperative pain relief as well as scales for the determination of pain intensity and patient satisfaction was explained. After informed consent, 120 patients were assigned to one of four groups, based on a computer-generated randomization table (http://www.randomization.com/) (Figure 1).

The four study groups were placebo, parecoxib, metamizole, and paracetamol (Table 1). Since normal saline used as placebo and paracetamol for IV infusion are provided in 100 mL solution, the other drugs were dissolved in 100 mL normal saline and given via IV infusion over 15 min. In all groups 10 min before extubation, 2 mg piritramide (Dipidolor, Janssen-Cilag) was injected concomitant to the tested medications. In the postoperative period, piritramide was offered in the form of a patient-controlled analgesia by means of a PCA pump as an electronically steered syringe pump.

The study solutions were prepared by one of the researchers who was not involved in the intraoperative and postoperative treatment of these patients, so the drugs could not be recognized by the anesthesiologists administering them and collecting the data. The observation time extended over a period of 24 hours after surgery. However, to ensure patient safety, a sealed opaque envelope containing the randomized treatment assignment was kept with each patient in the operating room and the ward to permit immediate unmasking in case of an emergency making this step necessary.

For premedication, midazolam 7.5 mg was administered orally 60 min before the surgical procedure. On arrival in the operating room, standard monitors were applied and a crystalloid infusion was started after placing an 18-gauge catheter in the nondominant hand for fluid administration intraoperatively. A second 18 G catheter in the other hand was used for the administration of anesthetic drugs and removed upon discharge from the postanesthesia care unit (PACU). After preoxygenation and precurarization, anesthesia was induced with 2 mg/kg propofol intravenously, followed by 1–1.5 mg/kg BW suxamethonium chloride to facilitate endotracheal intubation. Anesthesia was maintained with a supplemental infusion of 3–6 mg/kg/h propofol and 3–10.5 *μ*g/kg/h remifentanil for an adequate depth of anesthesia with mean arterial pressure and heart rate within 20% range of preoperative values.

Fifteen minutes before the expected end of surgery, each patient was treated according to the list of randomization (Table 1). The preprogrammed PCA equipment (Master PCA, Fresenius Vial Infusion Technology, Brézins, France) was provided with a 50 mL disposable syringe, and 45 mg piritramide in 45 mL saline solution was prepared for each patient. The PCA administered boluses of 2 mL (2 mg piritramide) with a lock-out interval of 10 min and a maximal volume of 30 mL in 4 h. A bolus of 2 mg piritramide was first injected 10 min prior to the extubation in the operating room.

Thereafter, the patients were directly transferred to the PACU, where further clinical observations were done by an independent, blinded observer who was unaware of the administered study drugs. On arrival, postoperative pain was treated by self-administration of small doses of IV piritramide using the PCA pump already mentioned and patients were asked every 2 hours for the first 6 hours and afterwards once every 6 hours to quantify their pain experience at rest on a visual analog scale (VAS) between 0 and 10, with 0 representing no pain and 10 the worst imaginable pain. Likewise, pain relief was assessed by the patient on a 0–3 verbal rating scale (VRS) (0 = none, 1 = mild, 2 = moderate, 3 = complete). Patient satisfaction with the effectiveness of pain therapy was inquired at 6-hour intervals by using a 4-point scale which shows the verbal expressed satisfaction of assigned numerical values: 1 = poor, 2 = moderate, 3 = good, 4 = very  good. The cumulative piritramide consumption within 24 hours postoperatively was recorded at discharge from the recovery room and after 6, 12, and 24 hours on the display of the PCA pump.

Data were first entered into Excel 2000 and then imported into SPSS for Windows Version 15.0 (SPSS Inc. Chicago, Illinois, USA) for calculations. An estimated sample size indicated that 30 patients per group would give a *β*-risk of 80% at an alpha level of 0.05 for detecting a difference in piritramide consumption of at least 5.0 mg at 24 h after the operation with a standard deviation of 7.0 for each group in the preliminary test.

For examination of the normal distribution, the Kolmogorov Smirnov test was applied. One-way analysis of variance (ANOVA) in normal-distributed continuous variables and Kruskal-Wallis's test in non-normal-distributed or ordinal variables between the groups were used. If there was a statistically significant difference between groups as determined by one-way ANOVA post-hoc tests (Bonferroni) were used to confirm where the differences occurred between groups. If the Kruskal- Wallis's test value was significant at the 0.05 level, groupwise comparisons were performed using Mann-Whitney rank sum test for unpaired data. Significant Mann-Whitney values were Bonferonni corrected with the factor for the number of comparisons resulting in a significance level set at *P* < 0.008. Categorical data were analyzed using *χ* ^2^ or Fisher's exact test as appropriate. Differences were judged significant at *P* < 0.05.

## 3. Results

The four groups were similar with respect to age, body mass index (BMI), ASA physical status, total remifentanil consumption, and duration of anesthesia (Table 2). The type and length of the arthroscopic surgical procedures were equally distributed in all 4 groups under the routine use of a tourniquet.

**Figure 2:**
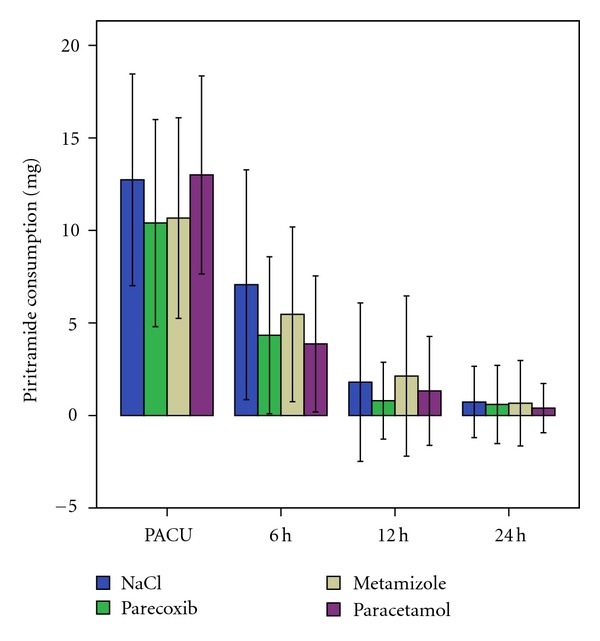
Incremental piritramide consumption in mg (mean and standard deviations) in the four groups over 24 hours postoperatively after arthroscopic knee surgery. There is no significant difference between the groups.

**Figure 3:**
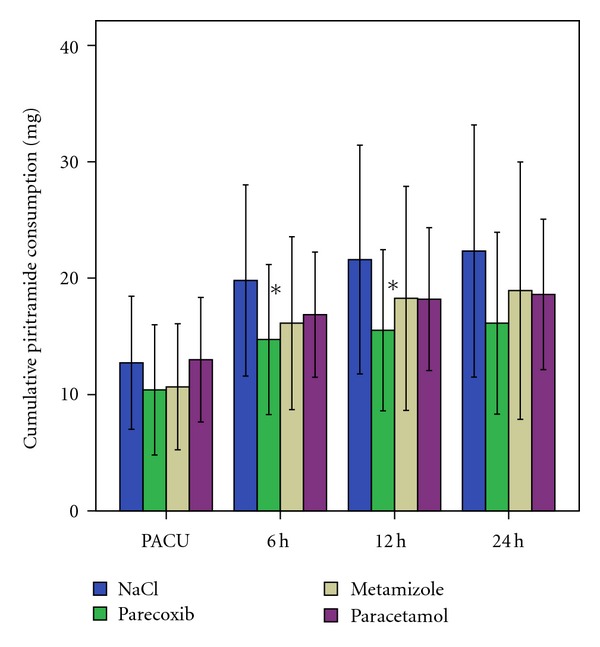
Cumulative piritramide consumption in mg (mean and standard deviations) over 24 hours postoperatively after arthroscopic knee surgery. *Parecoxib versus placebo at 6 h (*P* = 0.033) and at 12 h (*P* = 0.032).

The incremental piritramide consumption showed no significant differences at all times between the four groups (Figure 2). Upon arrival in the PACU, piritramide consumption was high in all groups. However, piritramide was no longer required in most patients at 24 h after surgery. Cumulative piritramide consumption is presented in Figure 3. Patients who received parecoxib used significantly less piritramide than those with placebo in intragroup comparisons at 6 h (*P* = 0,033) and 12 h (*P* = 0,032) after surgery. Apart from signs of mild nausea and vomiting, no further drug reactions were observed.

**Figure 4:**
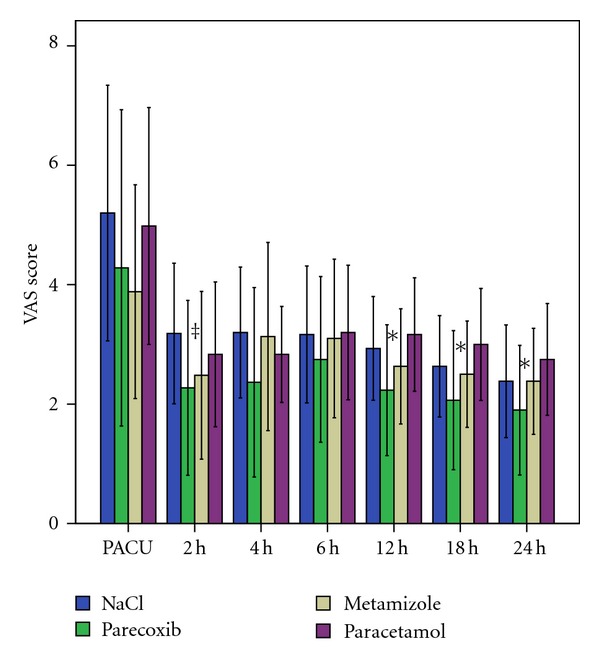
Visual analog scale (VAS, mean and standard deviations) over 24 hours postoperatively ^‡^ *P* = 0.006 NaCl versus parecoxib; *parecoxib versus paracetamol at 12 h (*P* = 0.002), at 18 h (*P* = 0.001), and at 24 h (*P* = 0.003).

VAS pain scores for all groups are presented in Figure 4. At discharge from the PACU, 2 hours after admission, VAS scores dropped in all groups and were significantly lower in the parecoxib group as compared to the placebo group (*P* < 0.006). Further significant differences between the groups were found at 12, 18, and 24 h after surgery. VAS scores were lowest in the parecoxib group at all measuring times.

In the PACU, satisfaction of the patients assessed on the 4-point scale was moderate and improved with time after surgery (Table 3). Satisfaction was statistically significantly higher in the parecoxib group compared with the metamizole and paracetamol groups at 6 and 12 h and with the paracetamol group at 24 h. The pain relief scores showed no statistically significant differences.

## 4. Discussion

Our findings show that pain was most intense immediately after recovering from remifentanil-based anesthesia for arthroscopic knee surgery and subsequently declined to low levels in all groups within 24 h after surgery. The early intense pain might be partly explained by a bolus dose of 2 mg piritamide with a lock-out time of 10 min which has been routinely prescribed in Germany [14]. Such smaller bolus doses with a short lock-out time might reduce piritramide consumption by enabling the patient to titrate analgesic effect more effectively; however, they obviously do not reduce opioid related side effects [16]. A background infusion in our study was not provided due to a possible increased risk of respiratory depression [7]. Furthermore, remifentanil-based anesthesia has been shown to be associated with postoperative hyperalgesia, even after a short-term exposure [29, 30], a fact which might have contributed to the overall pain in our patients. A significant difference in remifentanil consumption between the four groups was not found in our study.

A significant reduction in cumulative piritramide consumption was only shown in the parecoxib group compared to the NaCl group at 6 and 12 hours following arthroscopic knee surgery under general anesthesia. These results are in accordance with the data published in 2006, where in a 24 h study, cumulative opioid consumption was significantly reduced in the celecoxib group compared with the placebo group at 10 to 12 hours in patients undergoing ambulatory arthroscopic knee meniscectomy [31]. After the immediate postoperative period in the PACU, cumulative piritramide consumption in both paracetamol and metamizole groups also remained lower during the recording times of 24 hours after surgery as compared to the placebo; however, this was statistically not significant. Previously published systematic reviews and meta-analyses described opioid-saving effects [5, 9, 10]. A reason for the missing clear-cut opioid sparing effect in metamizole and paracetamol groups might be due to the non equivalent doses of these nonopioid analgesics administered in our study. We used 1 g metamizole and 1 g paracetamol three times daily (TID), whereas the maximum dose recommended by the manufacturer is 1 g four times daily (QID). In contrast, 40 mg parecoxib twice a day is the standard dosage recommended by the manufacturers for IV application in adults given each 12 hours. In a similar study in patients undergoing abdominal hysterectomy, we also did not find a significant difference in regard to opioid-sparing effect by administering additional non-opioid analgesics while VAS scores were significantly lower in the paracetamol and parecoxib groups at 6 h after surgery [13]. Any further benefits were marginal and statistically not significant. However, a lack of statistical significance on postoperative cumulative piritramide consumption was also found in different surgical procedures when parecoxib was given 40 mg twice daily and metamizole and paracetamol 4 g daily were administered [14, 15, 28, 32]. In a further newly published study, both parecoxib (80 mg/24 h) and paracetamol (5 g/24 h) effectively reduced postoperative opioid requirements after thyroid and parathyroid surgery [33]. These findings may show that the results of investigations are depending on the type of surgery performed and the dosages used and could be an incentive for other researchers to reevaluate different dosages of non-opioid analgesics in patients undergoing arthroscopic knee surgery. Other studies also evaluated the role of timing of analgesia, a point, we did not evaluate in our study: the preoperative administration has been reported to be equally effective to its postoperative use [34], or rather superior to it [31].

Apart from the statistically significant superiority of parecoxib in combination with piritramide given over a PCA after arthroscopic knee surgery, also the VAS pain scores were significantly lower in the parecoxib group as compared to placebo at discharge from the PACU, 2 hours after admission, and between parecoxib and paracetamol at 12, 18 and 24 h after surgery. However, VAS scores after 12 h were all ≤3, thus implying low pain intensity without the need for piritramide in most patients. Therefore, it might be difficult to demonstrate an additional benefit with an analgesic when baseline pain was already low. No drug reactions apart from mild nausea and vomiting, which were treated with antiemetics, occurred in our study.

A limitation of this study is that the trial was concluded in 2007 and that postoperative pain management may have changed from 2007 until now, which limits the generalizability of our findings. In addition, we used drugs which are not available in all countries. Also, we did not use the standard doses for metamizole and paracetamol recommended by the manufacturers. We had compared subtherapeutic doses for metamizole and paracetamol which are not equivalent to parecoxib. Furthermore, pain intensity was evaluated only at rest, and movement-related pain relief was not included in this study. In addition, the results might have been different if nonopioid analgesics had been given prior to surgery as preemptive analgesics. A comparison of the combined use of different drug classes (NSAID and paracetamol) given simultaneously as part of a multimodal treatment as in other studies might be worthwhile.

## 5. Conclusions

There was statistically significant opioid-saving effect only by administering parecoxib. The high pain score in PACU in all groups immediately after recovering from remifentanil-based anesthesia would be prevented if a local anesthetic solution was administered intra-articularly in all groups as part of a multimodal analgesic approach.

## Figures and Tables

**Table 1:** Patient groups (30 in each group) and treatment with normal saline (NS) and/or drug.

Group treatment	15 min prior to extubation	8 h postop.	12 h postop.	16 h postop.	24 h postop.
Placebo	NS	NS	NS	NS	NS
Parecoxib	40 mg	NS	40 mg	NS	NS
Metamizole	1 g	1 g	NS	1 g	NS
Paracetamol	1 g	1 g	NS	1 g	NS

**Table 2:** Demographic and patient-referred data of the investigated four groups. Values are mean (SD) or number of patients as appropriate.

Group	Placebo (*n* = 30)	Parecoxib (*n* = 30)	Metamizole (*n* = 30)	Paracetamol (*n* = 30)	*P* value
Sex					
Female	15	12	12	11	
Male	15	18	18	19	
Age (yr)					n. s.
Mean and SD	47.9 ± 11.8	48.3 ± 14.2	43.8 ± 13.7	44.5 ± 15.1	
BMI (kgm^−2^)					n. s.
Mean und SD	27.3 ± 3.3	27.0 ± 4.8	27.2 ± 4.7	28.1 ± 5.6	
ASA physical status					n. s.
II/III	26/4	20/10	25/5	25/5	
Total remifentanil consumption (mg)	0.66 ± 0.18	0.58 ± 0.15	0.64 ± 0.18	0.68 ± 0.13	n. s.
Duration of anesthesia (min)	65 ± 14	66 ± 12	68 ± 11	71 ± 10	n. s.
Arthroscopic surgical procedures	9 meniscus repair 13 ligament reconstruction 8 cartilage/bony reconstruction	8 meniscus repair 13 ligament reconstruction 9 cartilage/bony reconstruction	8 meniscus repair 14 ligament reconstruction 8 cartilage/bony reconstruction	9 meniscus repair 13 ligament reconstruction 8 cartilage/bony reconstruction	n. s.

There were no significant differences among the four groups.

**Table 3:** Patient satisfaction with the effectiveness of pain therapy within 24 hours after arthroscopic knee surgery.

Group	Placebo (*n* = 30)	Parecoxib (*n* = 30)	Metamizole (*n* = 30)	Paracetamol (*n* = 30)
PACU	2.4 ± 0.7	2.7 ± 0.7	2.6 ± 0.6	2.5 ± 0.6
After 6 h	2.9 ± 0.6	3.4 ± 0.6*	2.9 ± 0.6	2.8 ± 0.6
After 12 h	3.1 ± 0.6	3.4 ± 0.5*	3.0 ± 0.7	3.0 ± 0.6
After 18 h	3.3 ± 0.5	3.4 ± 0.5	3.2 ± 0.6	3.1 ± 0.6
After 24 h	3.4 ± 0.5	3.6 ± 0.5**	3.2 ± 0.6	3.1 ± 0.6

Data are presented as mean ± SD. *P < 0.008 parecoxib versus metamizole and paracetamol; **P < 0.008 parecoxib versus paracetamol.
